# Challenges and Future Trends of Hepatocellular Carcinoma Immunotherapy

**DOI:** 10.3390/ijms231911363

**Published:** 2022-09-26

**Authors:** Alessandro Rizzo, Angela Dalia Ricci

**Affiliations:** 1 Struttura Semplice Dipartimentale di Oncologia Medica per la Presa in Carico Globale del Paziente Oncologico “Don Tonino Bello”, I.R.C.C.S. Istituto Tumori “Giovanni Paolo II”, Viale Orazio Flacco 65, 70124 Bari, Italy; 2 Medical Oncology Unit, National Institute of Gastroenterology, “Saverio de Bellis” Research Hospital, 70013 Castellana Grotte, Italy

Hepatocellular carcinoma (HCC) is one of the most common cancers worldwide [1,2]. The efficacy of immune checkpoint inhibitors (ICIs) in several tumor types has prompted a similar development in HCC patients [3,4]. Firstly, two PD-1 inhibitors, nivolumab and pembrolizumab, were approved by the United States Food and Drug Administration (FDA) following the early phase CheckMate 040 and KEYNOTE-224 trials [5,6]. However, the two confirmatory phase III trials—CheckMate 459 and KEYNOTE-240-compared nivolumab versus sorafenib as first-line treatment, and pembrolizumab versus placebo as second-line therapy, respectively—failed to meet their primary endpoints [7,8]. If ICIs alone as a first-line treatment have not achieved the desired effect, clinical trials evaluating combinatorial strategies involving ICIs and other anticancer agents, especially antiangiogenic agents, have produced more compelling results, marking a new era in HCC management [9,10,11,12,13,14].

The practice-changing phase III IMbrave150 trial compared the combination of the PD-L1 inhibitor atezolizumab plus bevacizumab versus sorafenib monotherapy in treatment-naïve patients with advanced HCC [15,16]; of note, the results of IMbrave150 have led to the approval of this immune-based combination given an unprecedented median overall survival (OS) of 19.2 months compared with 13.4 months for sorafenib monotherapy (Hazard Ratio [HR], 0.58; 95% Confidence Interval [CI], 0.42–0.79). Similarly, the study reported a median progression-free survival (PFS) benefit (6.9 months versus 4.3 months, respectively) and higher overall response rate (ORR) in patients treated with the immune-based combination. Based on these results, atezolizumab–bevacizumab is currently considered the new standard of care in front-line HCC and has been approved in several countries worldwide [17,18]. Similarly, other combinations have been tested and are currently being assessed. Among these, the recently published COSMIC-312 phase III trial compared the combination of atezolizumab plus cabozantinib versus sorafenib as first-line treatment for advanced HCC [19]. Although the results indicated a statistically significant benefit in median PFS in patients treated with atezolizumab–cabozantinib, no difference in OS was highlighted. In another phase II/III trial, ORIENT-32, the investigators compared the combination of the PD-1 inhibitor sintilimab plus a bevacizumab biosimilar (IBI305) versus sorafenib alone, reporting a statistically significant improvement in terms of median PFS and OS in patients treated with the immune-based combination [20]. 

The role of another immune-based combination including two ICIs, the PD-L1 inhibitor durvalumab and the anti-CTLA-4 antibody tremelimumab, has been explored in the HIMALAYA trial (Figure 1) [21,22,23]. In this open-label, multicenter, phase III study, median OS was 16.4 months in patients receiving durvalumab plus tremelimumab versus 13.8 months in the sorafenib monotherapy arm, whereas no significant differences were reported in median PFS. Thus, the results of the HIMALAYA trial support the use of this immune-based combination in this setting. In addition, beyond immunomodulatory antibodies, several other agents and immune-based treatments have been assessed and are currently under evaluation, including adoptive cell transfer (ACT), oncolytic virus therapy, and vaccines [24,25,26,27,28].

From this point of view, this Special Issue welcomes papers exploring the current state of the art and future perspectives in the immunotherapy of HCC. The Special Issue will aim to assess key open questions in HCC immunotherapy, including preclinical studies, novel immunotherapies and immune-based combinations, biomarkers of response, experimental therapies, real-world experience with immune checkpoint inhibitors, and several other topics.

## Figures and Tables

**Figure 1:**
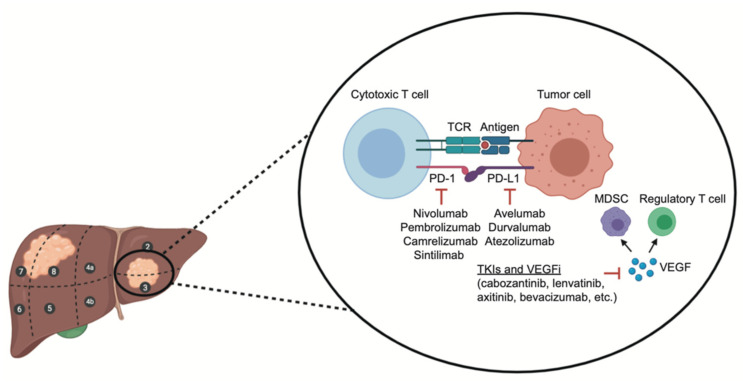
Schematic figure representing the synergistic activity of immune-based combinations (including double checkpoint blockade and immune checkpoint inhibitors plus antiangiogenic agents.

## Data Availability

Not applicable.
